# Kif5a Regulates Mitochondrial Transport in Developing Retinal Ganglion Cells In Vitro

**DOI:** 10.1167/iovs.64.3.4

**Published:** 2023-03-02

**Authors:** Satoshi Yokota, Sahil H. Shah, Emma Lee Huie, Runxia Rain Wen, Ziming Luo, Jeffrey L. Goldberg

**Affiliations:** 1 Spencer Center for Vision Research, Byers Eye Institute, Stanford University, Palo Alto, California, United States; 2 Kobe City Eye Hospital, Kobe, Hyogo, Japan

**Keywords:** retinal ganglion cells, mitochondria, motor protein

## Abstract

**Purpose:**

Axon transport of organelles and neurotrophic factors is necessary for maintaining cellular function and survival of retinal ganglion cells (RGCs). However, it is not clear how trafficking of mitochondria, essential for RGC growth and maturation, changes during RGC development. The purpose of this study was to understand the dynamics and regulation of mitochondrial transport during RGC maturation using acutely purified RGCs as a model system.

**Methods:**

Primary RGCs were immunopanned from rats of either sex during three stages of development. MitoTracker dye and live-cell imaging were used to quantify mitochondrial motility. Analysis of single-cell RNA sequencing was used to identify Kinesin family member 5A (Kif5a) as a relevant motor candidate for mitochondrial transport. Kif5a expression was manipulated with either short hairpin RNA (shRNA) or exogenous expression adeno-associated virus viral vectors.

**Results:**

Anterograde and retrograde mitochondrial trafficking and motility decreased through RGC development. Similarly, the expression of Kif5a, a motor protein that transports mitochondria, also decreased during development. Kif5a knockdown decreased anterograde mitochondrial transport, while Kif5a expression increased general mitochondrial motility and anterograde mitochondrial transport.

**Conclusions:**

Our results suggested that Kif5a directly regulates mitochondrial axonal transport in developing RGCs. Future work exploring the role of Kif5a in vivo in RGCs is indicated.

In developing neurons, adequate organelle transport to the sites of axonal branching, synapse formation, and growth cones is essential for maintaining metabolic support. In projection neurons, such as retinal ganglion cells (RGCs), areas of high energy consumption can be far removed from the soma, further highlighting the importance of axonal transport. Although embryonic and early postnatal RGCs continue to grow and extend their axons, they become unable to rapidly extend axons after postnatal day 5 (P5).^1^ While a variety of transcription factors, including Kruppel-like factors^2^ and STAT3,^3^ as well as growth factors like BDNF and CNTF^4^ have been shown to regulate this transition in the maturation of RGCs, the importance of changes in axonal transport of cargo through development is not fully understood. It has previously been shown that anterograde transport of organelles and neurotrophic factors is necessary for maintaining RGC function and survival,^5^^–^^7^ with mitochondrial axonal transport in particular being essential for neuronal health.^8^^,^^9^ Although axonal growth and intracellular signaling are known to trigger mitochondrial movement,^10^^,^^11^ the mechanism through which cellular changes regulate mitochondrial movement is not clear. Therefore, we hypothesized that changes in the anterograde axonal transport of mitochondria could be an important factor in the developmental switch in intrinsic capacity for rapid axon growth during RGC development.^12^

Mitochondria move along microtubule and actin tracks, mediated by motor proteins. Long-distance anterograde and retrograde fast axonal transport of mitochondria is mediated by kinesin and dynein, respectively.^11^^,^^13^^,^^14^ In particular, Kif1b and the Kinesin I family (Kif5a, Kif5b, Kif5c) have been shown to be involved in mitochondria transport in neurons.^15^ Among them, Kif5a is neuron specific and highly expressed in the central nervous system, and it has been implicated in neurodegenerative diseases such as amyotrophic lateral sclerosis (ALS) and, recently, RGC degeneration after optic nerve injury.^16^^,^^17^ Among the other Kinesin I family members, Kif5b is ubiquitously expressed, and Kif5c is enriched in motor neurons.^18^ During axonal trafficking, mitochondria can switch between anterograde and retrograde transport or remain stationary.^19^

Based on these data, we hypothesized that changes in expression of a member of the Kinesin I family might underlie mitochondrial dynamics during RGC development. We examined mitochondrial axonal transport in primary rat RGCs in culture, at different developmental stages and then after manipulating Kif5a expression. Here we present data demonstrating that Kif5a plays a major role in the axonal shuttling of mitochondria through development.

## Methods

### Ethics

All animal experiments conformed to the ARVO statement for the Use of Animals in Ophthalmic and Vision Research and were reviewed and approved by the Institutional Animal Care and Use Committee and the Institutional Biosafety Committee of Stanford University.

### RGC Purification and Culture

RGCs from E18, P2, and P9 rats (Sprague-Dawley) were purified by sequential immunopanning using an anti-Thy1 antibody to 99.5% purity, as previously described.^20^ Briefly, purified RGCs were cultured on poly-D-lysine (10 µg/mL; Sigma-Aldrich, St. Louis, MO, USA) and laminin (2 mg/mL; Telios/Gibco, NY, USA) in a dish (Ibidi, Munich, Germany; cat. 81156, µ-Dish 35 mm, high) with defined serum-free media: Neurobasal (Life Technologies, Carlsbad, CA, USA) supplemented with penicillin-streptomycin (100 U/mL; Sigma-Aldrich), insulin (5 µg/mL; Sigma-Aldrich), sodium pyruvate (1 mM; Sigma-Aldrich), L-glutamine (1 mM; Sigma-Aldrich), triiodothyronine (T3; 40 ng/mL; Sigma-Aldrich), N-acetyl cysteine (5 mg/mL; Sigma-Aldrich), B-27 Supplement (Gibco), Sato Supplement (1 µg/mL transferrin, 1 µg/mL BSA, 2 nM progesterone, 0.16 µg/mL putrescine, and 0.4 ng/mL sodium selenite), brain-derived neurotrophic factor (BDNF; 50 ng/mL; Peprotech, Rocky Hill, NJ, USA), CTNF (10 ng/mL; Peprotech), forskolin (5 nM; Sigma-Aldrich), and basic fibroblast growth factor (10 ng/mL; Peprotech). RGCs were cultured in 37°C, 10% CO_2_, for 5 days in vitro before imaging.

### Quantitative RT-PCR

Total RNA was isolated from rat RGCs of different ages using the RNeasy Microarray Tissue MiniKit (Qiagen, Germantown, MD, USA). RNA was then reverse transcribed using the iScript cDNA Synthesis Kit (Bio-Rad, Hercules, CA, USA). Quantitative RT-PCR was performed on a CFX Connect Real-Time PCR Detection System (Bio-Rad) using the Taqman primers for the rat Kif5a gene (cat. 4448892; Thermo Fisher Scientific, Waltham, MA, USA). The thermocycler was programmed as 94°C for 5 minutes, followed by 40 cycles of 94°C for 30 seconds, 56°C for 30 seconds, and 72°C for 60 seconds. GADPH was used as an internal control using TaqMan primers for the rat GAPDH gene (cat. 4453320; Thermo Fisher Scientific). All reactions were performed in technical triplicates, with experimental duplicates.

### Single-Cell RNA Sequencing Data

Previously published data from Clark et al.^21^ (GEO: GSE118614) were reanalyzed using the Seurat package version 4.1.1 in R version 4.0.3 (The R Foundation for Statistical Computing, Vienna, Austria).

### Viral Vectors

Kif5a expression was manipulated in culture with adeno-associated virus 2 (AAV2) viruses with an AAV2 capsid (AAV2/2). This capsid has a mutated tyrosine to phenylalanine in residues 444, 500, and 730, as described previously^22^ (gift of Dr. Sui Wang), for exogenous expression (AAV2.CMV.3xFLAG/mKif5a and AAV2.CMV.eGFP control) and for knockdown (AAV2.U6.shKif5a.CMV.mCherry and AAV2.U6.scramble.CMV.mCherry control).

### Time-Lapse Imaging of Mitochondrial Axonal Transport in RGCs

To image mitochondrial axonal transport, cultured RGCs were either nontransduced or transduced with the viruses above on day 1. After 5 days, mitochondria were labeled with 50 nM MitoTracker Deep Red FM (Invitrogen, Carlsbad, CA, USA) for 15 minutes and imaged every 1.5 seconds for 3 minutes by confocal microscope with incubation (LSM880; Carl Zeiss Meditec, Dublin, CA, USA) over a wide field of view. From the time-lapse images, we generated kymographs using the ImageJ (National Institutes of Health, Bethesda, MD, USA) Multiple Kymograph plug-in for ImageJ submitted by J. Rietdorf and A. Seitz (European Molecular Biology Laboratory, Heidelberg, Germany) to categorize mitochondrial state and calculate speed of the transport as described previously.^23^ In all time courses, mitochondria with a total absolute distance of >10 µm defined over a period of 3 minutes were included as motile. Any mitochondria 50 µm from the cell body or the end of the neurite were excluded. Although it is not possible to definitively differentiate between axons and dendrites at this stage, the longest neurite and main branch were chosen as the candidate axon. Additionally, cells were plated at low density to avoid contact between cells, and neurites touching neighboring cells were excluded from analysis.

### Immunocytochemistry

Cultures were fixed using prewarmed 37°C 4% paraformaldehyde. Cultures were washed with PBS, then blocked and permeabilized with 5% normal goat serum and 0.02% Triton X-100 in antibody buffer (150 mM NaCl, 50 mM Tris base, 1% BSA, 100 mM L-lysine, 0.04% Na Azide, pH 7.4) for 1 hour at room temperature to reduce nonspecific binding. Samples were incubated overnight at 4°C in antibody buffer containing primary antibody (Ms anti-FLAG, F1804, 1:1000; Sigma-Aldrich), washed with PBS, incubated in antibody buffer containing secondary antibodies for 1 hour at room temperature, and washed with PBS. Cultures were left in PBS for imaging; cultures were reimaged using fluorescence microscopy (Observer.Z1; Carl Zeiss Meditec, Dublin, CA, USA).

### Selection Criteria

For neurons transduced with FLAG-tagged Kif5a, selection of positively transduced cells was not possible during time-lapse live-cell imaging. Instead, neurons were time-lapsed imaged for mitochondrial movement, then fixed and stained for FLAG as described above. The same regions of the wells were then imaged using epifluorescence microscopy. The FLAG-positive cells were identified and correlated to the live-cell microscopy videos. Only FLAG-positive cells were used for subsequent kymograph analysis. For all other samples, fluorescent tags in the viruses were used to identify positive cells.

### Western Blotting

Embryonic hippocampal and cortical neurons were cultured as described previously.^24^ Briefly, neurons were seeded at a density of 200,000 cells/well in 6-well tissue culture dishes (Falcon, Glendale, AZ, USA) precoated with poly-D-lysine (0.1 mg/mL; Sigma-Aldrich) and cultured in defined medium of Neurobasal, l-glutamine (2 mm), penicillin-streptomycin, and B27 supplement (1:50; all from Thermo Fisher Scientific). On day 3, adeno-associated viruses (AAVs) were transduced at a multiplicity of infection of 100,000. On day 7, cultures were rinsed with Dulbecco's phosphate-buffered saline (DPBS) and subsequently lysed in 500 µL RIPA with protease inhibitor cocktail followed by sonication. Lysate was clarified with centrifugation at 10,000 × *g* for 10 minutes. Samples were boiled for 5 minutes in SDS sample buffer and run on 4% to 20% TGX gels (Bio-Rad). Then, 0.2-µm nitrocellulose membranes were probed with rabbit anti-Kif5a (ab5628, 1:2000; Abcam, Cambridge, MA, USA), mouse anti-FLAG (F1804, 1:1000; Sigma-Aldrich), mouse anti-mCherry (ab125096, 1:5000; Abcam), or mouse anti-GFP (ab1218, 1:5000; Abcam) diluted in 5% milk with 0.2% Tween-20 in tris-buffered saline (TBST).

### Statistical Analysis

All statistical comparisons were done as described in the figure legends. A portion of the data presented later in Figures 3a and 3b (part of the E18 viral manipulation data) was given as preliminary data in a previous publication.^17^ Since that publication, further experimental replicates have been used and merged with the original data for this article. For binary data, a binomial logistic regression model was created to estimate variance and significance. Individual data points represent averages per individual RGC culture across multiple wells consisting of multiple neurons. For continuous data, confidence intervals are calculated per mitochondria. Every condition used in the article consists of >1000 individual quantified mitochondria.

## Results

### Mitochondrial Axonal Transport Decreases as RGCs Mature

**Figure 1.**
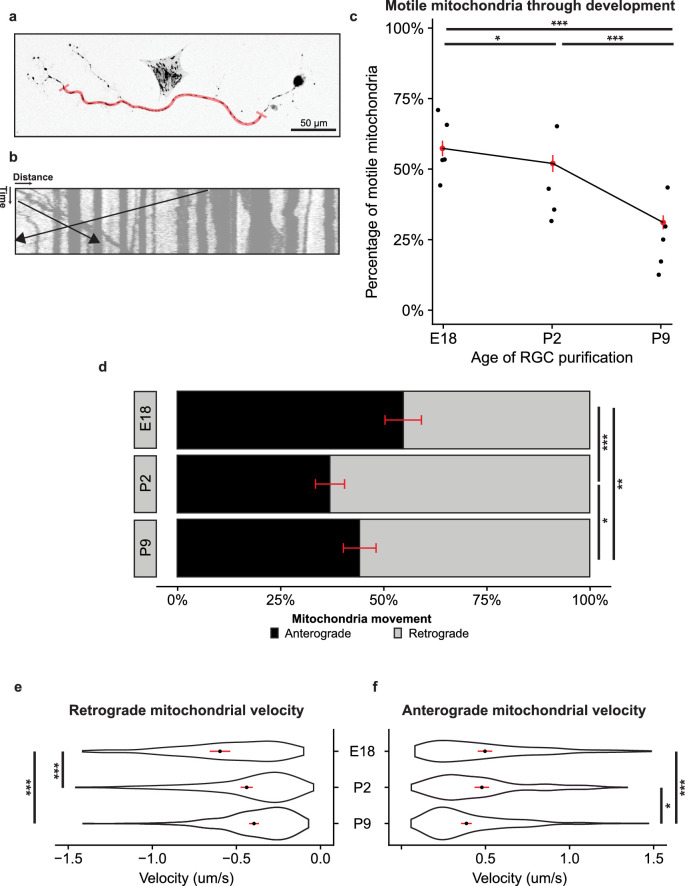
Anterograde mitochondrial trafficking decreases through RGC development. (**a**) Representative image of live-cell imaging of MitoTracker-labeled mitochondria in an E18 immunopanned RGC. The region of the axon used for quantification is highlighted in *red*. In total, 50 µm from either the soma or axon terminal was excluded. *Scale bar*: 50 µm. (**b**) Kymograph representation of mitochondrial motility. Distance is measured horizontally, and time is measured vertically. The *black lines* represent retrogradely and anterogradely moving mitochondria. (**c**) Percentage of motile mitochondria per total mitochondria by RGC developmental time point decreased in transport with increasing age. Each point represents average percent motility by experimental replicate, with a weighted mean in *red*. Confidence intervals represented by a *red line*. Binomial logistic regression across all mitochondria sampled, E18 to P2, *P* = 0.012; P2 to P9, *P* < 0.001; E18 to P9, *P* < 0.001. E18, *n* = 1199/15/5 (total mitochondria/total RGCs/total experimental replicates); P2, *n* = 1027/17/4; P9, *n* = 1300/17/5. (**d**) Fraction of anterogradely moving mitochondria (*black*) compared to retrogradely moving mitochondria (*gray*) decreased through RGC development. Binomial logistic regression, E18 to P2, *P* < 0.001; P2 to P9, *P* = 0.02; E18 to P9, *P* = 0.001. (**e**) Retrograde mitochondrial velocity decreased across developmental time points. Violin plot showing density, mean (*black dot*) with 95% confidence interval (*red line*). Tukey multiple comparisons. E18 to P2, *P* < 0.001; P2 to P9, *P* = 0.90; E18 to P9, *P* < 0.001. (**f**) Anterograde mitochondrial velocity decreased across developmental time points. E18 to P2, *P* = 0.20; P2 to P9, *P* = 0.013; E18 to P9, *P* < 0.001. *P < 0.05, **P < 0.01, ***P < 0.001.

To ask how mitochondria transport changed in RGCs throughout development, we used MitoTracker Deep Red FM (M22426; Thermo Fisher) to label mitochondria and performed time-lapse live-cell imaging to record mitochondria axonal transport in immunopanned RGCs from rats in different stages of development, embryonic day 18 (E18), postnatal day 2 (P2), and late postnatal day 9 (P9). We excluded areas of the axon 50 µm from either the soma or the axon terminal (Fig. 1a). To quantify mitochondria axonal transport direction and velocity, we used the live-cell imaging data to generate kymographs, the two-dimensional representation of mitochondrial position versus time^25^^,^^26^ (Fig. 1b).

We first asked how the balance of motile versus nonmotile mitochondria changed through development. We hypothesized that mitochondrial shuttling would be more active during times of neurite extension. Indeed, we found that the percentage of motile mitochondria significantly decreased from late prenatal through early postnatal RGCs (Fig. 1c). We then dissected the motile fraction further and found a significant shift away from anterograde transport during this period (Fig. 1d). Finally, we quantified the velocity of motile mitochondria across development. Within the retrograde portion, we found a significant decrease in velocity from E18 to P2, which remained decreased at P9 (Fig. 1d). Conversely, anterograde velocity was not significantly changed from E18 to P2 but was decreased by P9. Together, these data suggest that mitochondrial motility, the anterograde fraction of motile mitochondria, and the velocity of anterograde mitochondria all decrease through the critical period of RGC development when axon growth slows.

### Kif5a Expression Decreases in RGCs Through Development

We next asked which anterograde motor may regulate mitochondrial movement during this period. As previous research has demonstrated the role of Kif5a, a kinesin heavy chain protein, in embryonic mitochondrial transport in RGCs,^17^ we hypothesized that the change in expression of specific isoform Kif5a in RGCs would complement the changes seen in anterograde mitochondrial motility.

**Figure 2.**
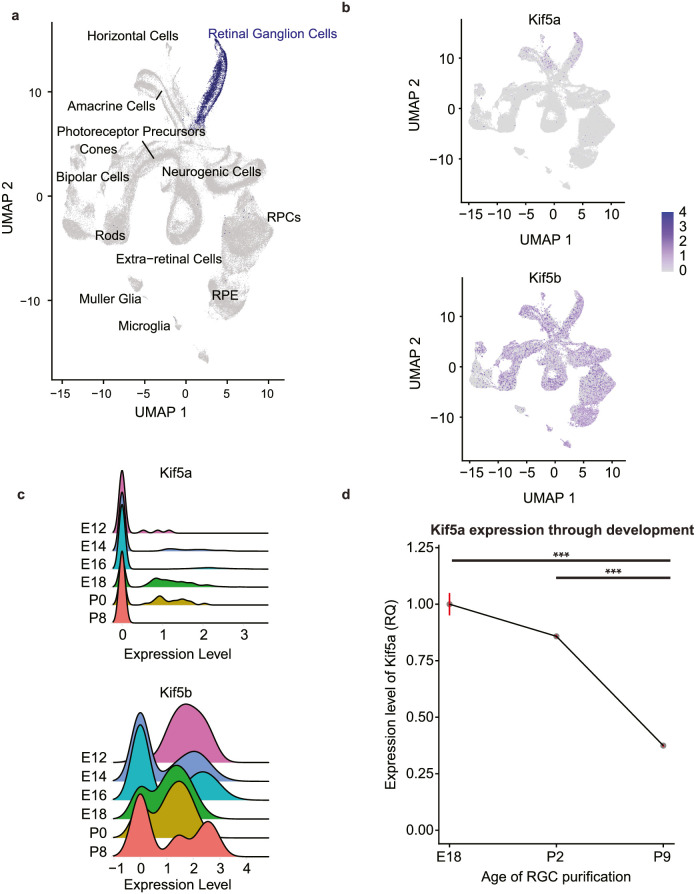
Kif5a expression decreases during RGC development. (**a**) UMAP dimension reduction plot of single-cell RNA sequencing of the developing retina (RGCs highlighted in *blue*), constructed with data from Clark et al.^21^ (**b**) Kif5a and Kif5b gene expression highlighted on UMAP plot (*blue*). (**c**) Kif5a and Kif5b gene expression over developmental time points within the identified RGC cluster. (**d**) Quantitative RT-PCR expression levels of Kif5a in RGCs isolated from rats decreased with age. All data were normalized to E18 Kif5a expression levels. E18 to P2, *P* = 0.053; E18 to P9, *P* < 0.01; P2 to P9, *P* < 0.01. *N* = 2. ***P < 0.001.

To address this question, we explored a publicly available retinal developmental single-cell RNA sequencing (sc-seq) database.^21^ First, we used a UMAP dimension reduction of all samples from E11 to P8, focusing on RGCs (Fig. 2a, RGCs highlighted). We probed this data set for two sister Kinesin I isoforms, Kif5a and Kif5b. Kif5a had lower expression levels across all samples but was relatively specific for RGC and amacrine cell populations. In contrast, Kif5b was ubiquitously expressed across the retina throughout development (Fig. 2b). After isolating the RGC cluster, we examined the expression profiles by age. Although dropout is a known problem with low expression genes in sc-seq data sets,^27^ Kif5a increased in expression through embryonic development and decreased after P0. In contrast, Kif5b expression did not vary through development (Fig. 2c). Together, the sc-seq data support Kif5a as an RGC-enriched motor protein whose expression pattern matches the change in anterograde mitochondrial transport.

To validate these findings in our model system and match our developmental time points, we performed quantitative PCR for Kif5a in E18, P2, and P9 immunopanned RGCs. Kif5a expression significantly decreased between P2 and P9, confirming the sc-seq data (Fig. 2d). Thus, the decrease in Kif5a expression parallels the decline in RGCs’ anterograde transport of mitochondria.

### Kif5a Knockdown Decreases Anterograde Transport in RGCs

**Figure 3.**
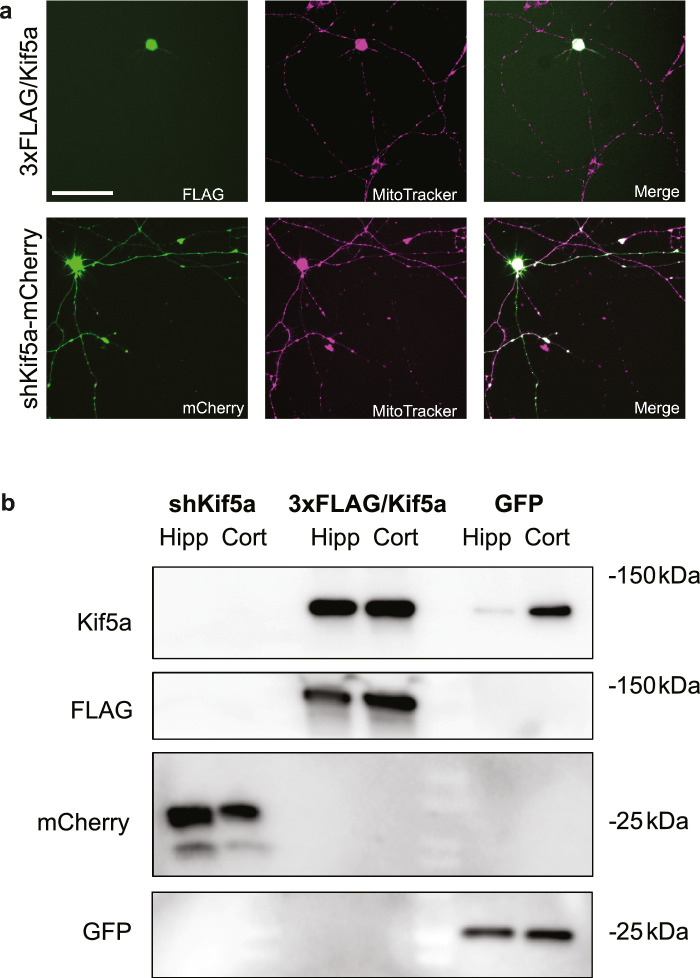
Validation of Kif5a expression and knockdown AAVs. (**a**) After transduction of cultured RGCs (E18 RGCs pictured) with either AAV2.CMV.3xFLAG/Kif5a (*top row*) or AAV2.CMV.shKif5a.U6.mCherry (*bottom row*), MitoTracker Deep Red (*middle column*) was used to visualize mitochondria. In exogenous expression experiments, RGCs were subsequently fixed and stained with anti-FLAG antibody (*top left*) to identify positively transduced cells, which were used as the selection criteria to quantify only positively transduced cells. In knockdown experiments, mCherry was imaged (*bottom left*) to identify positively transduced cells. Overlay images demonstrating detection of positively transduced cells for mitochondrial analysis (*right*). *Scale bar*: 100 µm. (**b**) Western blot validation of changes in Kif5a expression in two neuronal cell populations. Either hippocampal or cortical neurons were isolated from E18 pups and grown in culture. Either Kif5a knockdown (Kif5a KD), expression (Kif5a OE), or GFP control (GFP) transduced cells were probed for Kif5a, FLAG, mCherry, and GFP.

Given these findings, we reasoned that as Kif5a expression levels decreased with age in RGCs and possibly resulted in decreased mitochondrial axonal transport, manipulating Kif5a expression would alter mitochondrial transport dynamics throughout RGC development. We first developed and validated Kif5a targeting exogenous expression and knockdown viruses to manipulate anterograde transport (Fig. 3a). While relatively equal amounts of fluorescent or affinity tags were detected in each condition, levels of Kif5a were enhanced in both cell types in the overexpression condition and undetectable in the knockdown condition, even though baseline expression of Kif5a was variable (Fig. 3b). We then tested this hypothesis by knocking down Kif5a in RGCs of different ages and quantifying mitochondrial axonal transport. We used AAV delivery of short hairpin RNA (shRNA) against the Kif5a transcript with a fluorescent reporter, with a nontargeting scramble shRNA as a control. We then calculated the change in motility, anterograde and retrograde mitochondrial axonal transport ratio, and anterograde mitochondrial velocity in RGCs of different ages.

**Figure 4.**
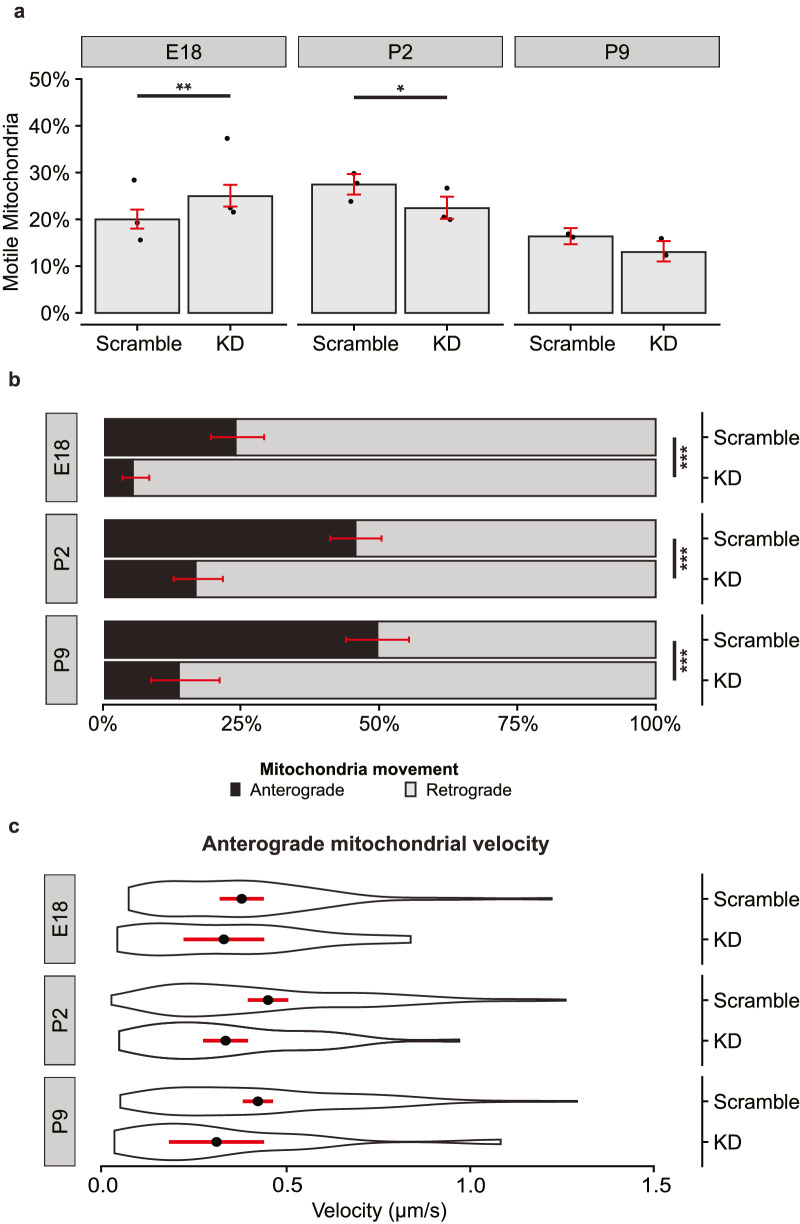
Kif5a knockdown disrupts anterograde mitochondrial transport in developing RGCs. (**a**) Fraction of motile mitochondria per developmental age and viral condition. Individual *dots* represent average motility per culture. *Bar height* represents weighted mean from all cultures. *Errors bars* and significance generated by binomial logistic regression. E18, *P* = 0.008; P2, *P* = 0.01; P8, *P* = 0.1. E18 scramble, *n* = 1496/19/3 (total mitochondria/total RGCs/total experimental replicates); E18 Kif5aKD, *n* = 1333/20/3; P2 scramble, *n* = 1585/20/3; P2 Kif5aKD, *n* = 1192/22/3; P9 scramble, *n* = 1780/17/2; P9 Kif5aKD, *n* = 914/14/2. (**b**) Fraction of anterograde (*black*) or retrograde (*gray*) mitochondria decreased in RGCs transduced with AAV-shRNA-Kif5a compared to AAV-shRNA-scramble. Fisher's exact test: E18, *P* < 0.001; P2, *P* < 0.001; P9, *P* < 0.001. *Error bars* (*red*) estimated by binomial logistic regression. (**c**) Velocity of anterogradely moving mitochondria did not change with AAV-shRNA-Kif5a compared to AAV-shRNA-scramble. Violin plot with mean (*black dot*) with 95% confidence interval (*red line*). *t*-test: E18, *P* = 0.47; P2, *P* = 0.06; P9, *P* = 0.09. *P < 0.05, **P < 0.01, ***P < 0.001.

We found a statistically significant but small effect on the fraction of motile mitochondria after viral transduction in E18 and P2 but not P9 RGCs (Fig. 4a). Of note, the proportion of motile mitochondria is decreased in all virally transduced samples compared to the naive cultured neurons seen in Figure 1, so to avoid the confound of transduction, we compared only transduced RGCs (scramble shRNA versus Kif5a shRNA) in this experiment. In contrast to this minimal effect on mitochondrial motility, we found that Kif5a knockdown significantly decreased the percentage of anterograde mitochondrial transport compared to retrograde transport in motile mitochondria throughout development (Fig. 4b). This effect also held true in the P9 cohort, which had lower levels of endogenous Kif5a. Interestingly, the velocity of the remaining anterogradely moving mitochondria was not significantly affected, although there was a borderline significant decrease in velocity in the P2 population (*P* = 0.06) (Fig. 4c). Together, these data indicate Kif5a expression is necessary, at least in part, for normal anterograde transport of mitochondria at all stages of RGC maturation.

### Exogenous Expression of Kif5a Increases Mitochondrial Anterograde Axonal Transport in RGCs

**Figure 5.**
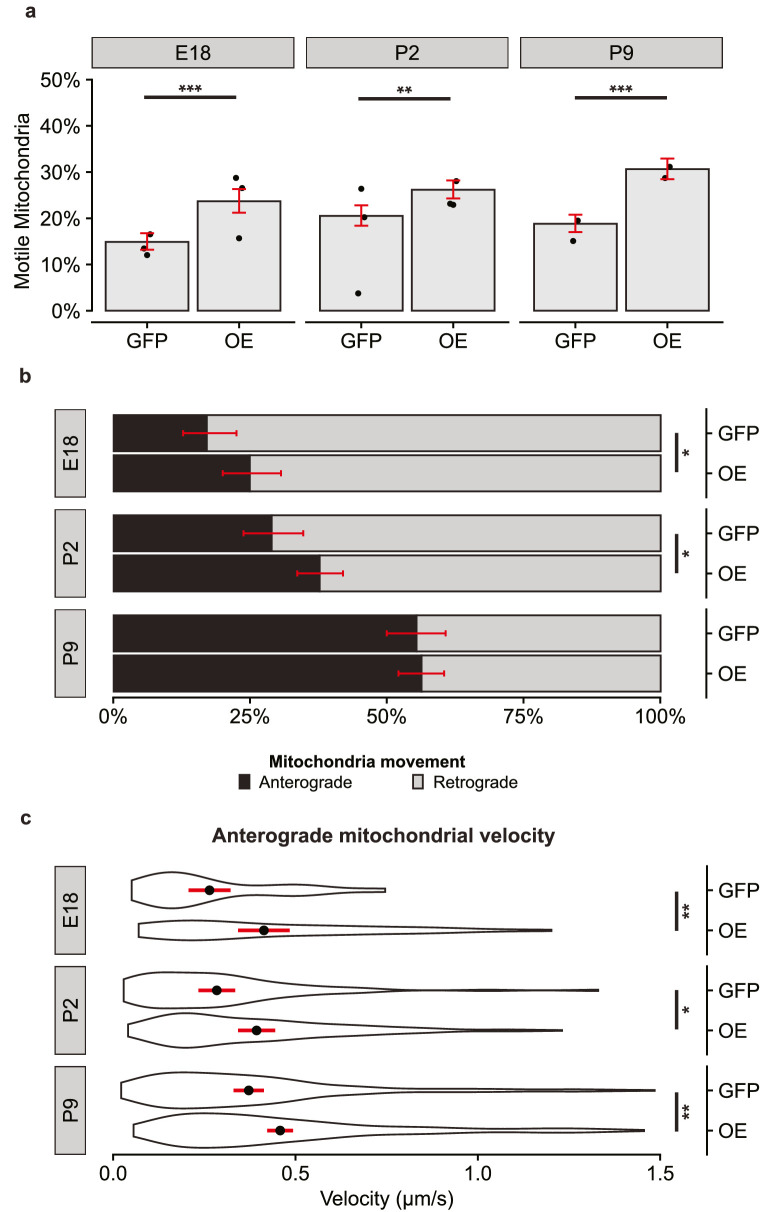
Exogenous expression of Kif5a enhances anterograde mitochondrial transport in developing RGCs. (**a**) Fraction of motile mitochondria per developmental age and viral condition. Individual *dots* represent average motility per culture. *Bar height* represents weighted mean from all cultures. *Errors bars* and significance generated by binomial logistic regression. E18, *P* < 0.001; P2, *P* = 0.001; P8, *P* < 0.001. E18 GFP, *n* = 1538/22/3 (total mitochondria/total RGCs/total experimental replicates); E18 Kif5aOE, *n* = 1069/13/3; P2 GFP, *n* = 1282/19/3; P2 Kif5aOE, *n* = 1967/27/3; P9 GFP, *n* = 1642/18/2; P9 Kif5aOE, *n* = 1648/17/2. (**b**) Fraction of anterograde (*black*) or retrograde (*gray*) mitochondria decreased in E18 and P2 RGCs transduced with AAV-Kif5a compared to AAV-GFP. Fisher's exact test: E18, *P* = 0.04; P2, *P* = 0.02; P9, *P* = 0.83. (**c**) Velocity of anterogradely moving mitochondria increased in RGCs transduced with AAV-Kif5a compared to AAV-GFP. Violin plot with mean (*black dot*) with 95% confidence interval (*black line*). *t*-test: E18, *P* = 0.005; P2, *P* = 0.015; P9, *P* = 0.003. *P < 0.05, **P < 0.01, ***P < 0.001.

Given the strong effect of Kif5a knockdown seen in all developmental RGC stages, we next used AAV vectors with either exogenous expression of Kif5a with a FLAG tag or GFP control. We hypothesized that increasing expression of Kif5a would be sufficient to shift the balance of mitochondrial trafficking to the anterograde direction. Indeed, we found that expressing Kif5a significantly increased the motile pool of mitochondria in all developmental time points (Fig. 5a). Again, baseline mitochondrial motility was reduced in these virally transduced RGCs compared to the naive neurons in Figure 1. We proceeded with direct comparisons between GFP- and Kif5a-expressing AAVs for analysis in these experiments. Surprisingly, while we found a significant increase in anterograde mitochondrial transport in the E18 and P2 RGCs, time points with high endogenous Kif5a expression, we did not detect a shift in P9 RGCs, a time point with lower endogenous Kif5a expression (Fig. 5b). We then asked if this exogenous expression affected the velocity of anterograde mitochondrial transport and found a significant increase at all three time points (Fig. 5c).

Taken together, these data suggest that exogenous Kif5a expression is directly tied to mitochondrial velocity and the fraction of motile mitochondria, as well as increases the ratio of anterograde/retrograde transport, at least embryonic and early postnatal RGCs in culture.

## Discussion

RGCs rely on the anterograde and retrograde shuttling of mitochondria, among other cargoes, down their axons. During development, areas of high energy utilization include branch points,^28^ growth cones,^29^ and nascent synapses.^30^ After maturity, mitochondrial transport continues to play a major role in maintaining healthy axons and synapses for continued transmission of information.^31^ In neurodegenerative diseases like glaucoma, some of the first signs of RGC dysfunction include changes in mitochondrial size, localization, and overall energy dynamics.^32^ Interestingly, several studies in multiple animal models have shown that increasing mitochondrial trafficking increases axonal regeneration after optic nerve injury.^33^^–^^35^ We now demonstrate that mitochondrial motility, anterograde transport, and velocity are all decreased through development, partly regulated by Kif5a function. As mitochondrial shuttling is a vital component of RGC axon extension both during development and as a candidate for regenerative therapies, further dissection of how mitochondrial dynamics change when RGCs transition after their developmental period of rapid axon growth may lead to new approaches for increasing functional, regenerated RGCs after injury.

Although Kif5a has previously been identified as a motor protein capable of anterograde mitochondrial transport,^17^^,^^36^^–^^38^ it was not known how Kif5a-dependent transport contributed to RGC maturation. It is now clear that a decrease in expression of Kif5a contributes to the decrease in mitochondrial shuttling. It is surprising that exogenous expression of Kif5a did not enhance the anterograde/retrograde transport ratio in P9 RGCs despite increases in the overall pool of motile mitochondria, suggesting an additional layer of transport regulation. Whether this regulation occurs at the level of adaptor proteins, Kif5a inhibition, or a compensatory increase in dynein activity is not known and will be the subject of future studies.

A limitation to this study is that these measurements were performed in RGCs purified into in vitro cultures. Although this model system allows careful dissection of RGCs’ intrinsic properties, these cultures lack the connectivity and support RGCs experience in vivo and thus may not fully reflect in vivo phenotypic complexity. This is even more true of P9 and older RGCs that are more difficult to immunopan and keep healthy in vitro. In addition, viral transduction is an added stressor to the health of these cells, reflected in the difference in motility between transduced and nontransduced cells. The baseline motility is reduced in virally transduced samples compared to naive controls, an important caveat when extending these results to in vivo biology. To account for this, we used direct comparisons with expression and knockdown controls to isolate the effects of Kif5a, acknowledging that the baseline in the control samples may be different than in nontransduced cells. Similarly, there may be differences in axon length between virally transduced samples and naive controls. While we controlled for major differences by excluding areas close to the soma or axon terminals, future work analyzing different axon lengths and distance from the soma will further expand our understanding of RGC axon mitochondrial dynamics. Another limitation of this study is a lack of direct live-cell imaging of Kif5a bound to mitochondria. Given the size of the Kif5a gene, it was not feasible to package a GFP-Kif5a-fusion protein in an AAV viral vector. Even with these limitations, the effects of Kif5a expression and knockdown were significant and provided direct evidence of Kif5a's role in mitochondrial transport. Future research should include direct imaging of Kif5a transport, an exploration of retrograde transport of mitochondria, and the effects of Kif5a manipulation in adult RGCs, including expanding prior work exploring Kif5a in RGCs in vivo.^17^

In addition to mitochondria, Kif5a is responsible for axonal transport of proteins and RNA to the axon and axon terminal.^39^ In recent years, mutations in Kif5a have been implicated in neurodegenerative diseases including Charcot-Marie-Tooth and ALS^40^^–^^42^; however, the exact mechanism of how Kif5a mutations cause these diseases remains unclear. Interestingly, the Kif5a knockout mouse is embryonic lethal, and motor neurons isolated from these knockout mice show decreased mitochondria transport speed in both anterograde and retrograde directions in culture.^43^ The full complement of Kif5a cargo, and in fact that of other kinesin motor proteins, is not fully understood. How this cargo specificity changes through development, such as when RGCs stop extending axons and start maturing, and in disease states, such as optic nerve injury or glaucoma, remains to be seen, but untangling the web of motor protein–dependent organelle, protein, and mRNA transport will guide therapeutic efforts to reestablish function cellular trafficking.
